# Effect of Two Anti-Fungal Treatments (Metrafenone and Boscalid Plus Kresoxim-methyl) Applied to Vines on the Color and Phenol Profile of Different Red Wines

**DOI:** 10.3390/molecules19068093

**Published:** 2014-06-16

**Authors:** Noelia Briz-Cid, María Figueiredo-González, Raquel Rial-Otero, Beatriz Cancho-Grande, Jesús Simal-Gándara

**Affiliations:** Nutrition and Bromatology Group, Analytical and Food Chemistry Department, Faculty of Food Science and Technology, University of Vigo, Ourense Campus, Ourense E-32004, Spain

**Keywords:** metrafenone, boscalid, kresoxim-methyl, color, phenolic profile, Graciano red wines, Tempranillo red wines

## Abstract

The effect of two anti-fungal treatments (metrafenone and boscalid + kresoxim-methyl) on the color and phenolic profile of Tempranillo and Graciano red wines has been studied. To evaluate possible modifications in color and phenolic composition of wines, control and wines elaborated with treated grapes under good agricultural practices were analyzed. Color was assessed by Glories and CIELab parameters. Color changes were observed for treated wines with boscalid + kresoxim-methyl, leading to the production of wines with less color vividness. Phenolic profile was characterized by HPLC analysis. Boscalid + kresoxim-methyl treatment promoted the greatest decrease on the phenolic content in wines.

## 1. Introduction

The main difficulty in growing grapes for wine is the fight against fungal diseases caused by fungi such as grey mold (*Botrytis cinerea*), powdery mildew (*Uncinula necator*) and downy mildew (*Plasmopara viticola*). Although practicing different traditional techniques it can be possible to minimize the incidence of these fungi during cultivation of the grape, the most effective means to combat them is the application of fungicides. With time, fungi can develop resistance to the most frequently applied fungicides, making it necessary to replace the traditionally used fungicides by others that include new generation active substances or new fungicides [1]. Several studies report that fungicides applied to vine may persist at trace levels in the grapes and thus be transferred to grape juice and ultimately to the wine [2,3,4], modifying the sensory quality of the final wine by causing changes in the fermentation kinetics and in the aromatic profile [2,5,6,7].

However, the effect of the presence of fungicide residues in grapes on the extraction of polyphenolic compounds during the winemaking process or their evolution during wine storage or aging remains almost completely unexplored. Some studies have confirmed that the phenolic composition of Monastrell red wines was altered by the presence of fungicide residues [4,8]. In fact, reductions in the anthocyanin content were found in wines obtained from grapes treated with famoxadone, fenhexamid and trifloxystrobin, while the hydroxycinnamic acid content decreased in the case of treatments with famoxadone, fluquinconazole, kresoxim-methyl and trifloxystrobin [4]. Thereby, the extraction of phenolic compounds during fermentation could be affected as a consequence of the presence of fungicide residues and this could originate problems in the stabilization of the wine color characteristics during storage.

The main objective of the present study was to evaluate the effect of some new fungicides (metrafenone, boscalid and kresoxim-methyl) on the accumulation of the major phenolic compounds in Tempranillo and Graciano red wines produced in La Rioja (N.E. Spain). For this, vineyards were treated with these fungicides under good agricultural practices. 

## 2. Results and Discussion

Tempranillo (T) and Graciano (G) are the most distinctive red grapes of La Rioja (N.E. Spain). The first one is the most characteristic grape variety of this region and is able to produce wines with long aging, very balanced in alcohol content, color and acidity [9]. On the other hand, Graciano is often used as a blending partner of Tempranillo-based wines due to its contribution to improve the color of T wines and to add aroma, tannins and acidity [10]. Although it is less usual, some wineries also produce monovarietal G wines [10]. Graciano presents a certain resistance to diseases such as downy mildew and powdery mildew, has low fertility, is late maturing and produces wines with considerable acidity and polyphenolic content, ideal for aging, with a very intense aroma. The color and phenolic profile of both varieties as well as the effect of fungicide treatments on these parameters were established in this study.

### 2.1. Influence of New Generation Fungicides Residues on the Color

The color of a red wine is closely related with the grape variety, degree of ripeness, time and temperature of maceration process. The color of a red wine can be established by using colorimetric indexes and the CIELab parameters. Regarding colorimetric indexes, if we compare T and G control wines (without fungicide treatments), T-Control showed a higher yellow color contribution (38%) and a higher tonality (77) in comparison to G-Control (% yellow and tonality of 35 and 62, respectively). However, T-Control showed a lower red color contribution (49%) and color intensity (0.5) than G-Control (% red and color intensity of 56 and 0.6, respectively). Regarding to CIELab space, T-Control wines showed lower chroma (C_ab_* = 29), higher lightness (L* = 70) and hue angle (h_ab_ = 4) than G-Control wines (whose values were 39, 67 and 1, respectively). All these results indicate that Graciano variety provides darker and colorful wines than the Tempranillo variety. These results are in agreement with those of other authors [9]. 

**Figure 1:**
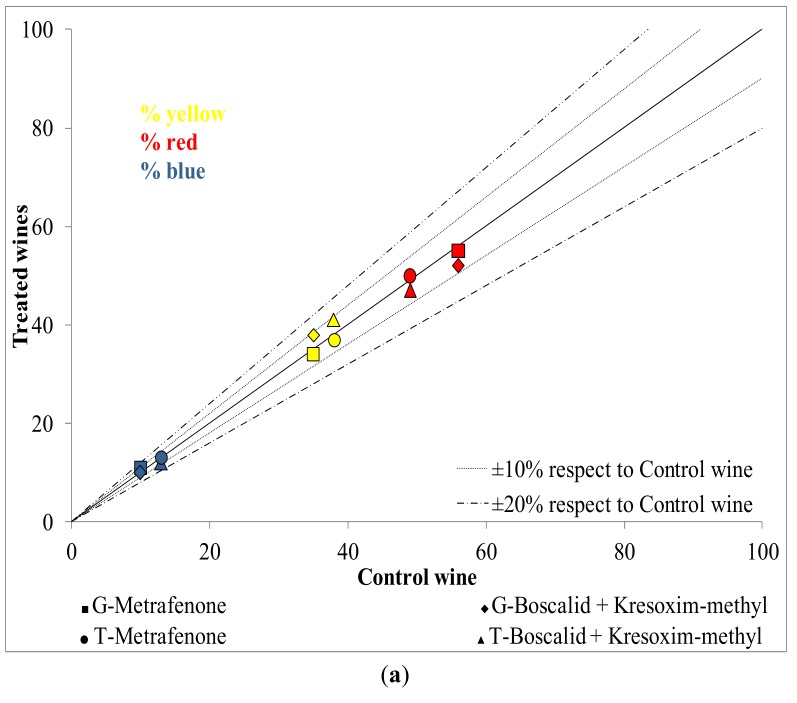

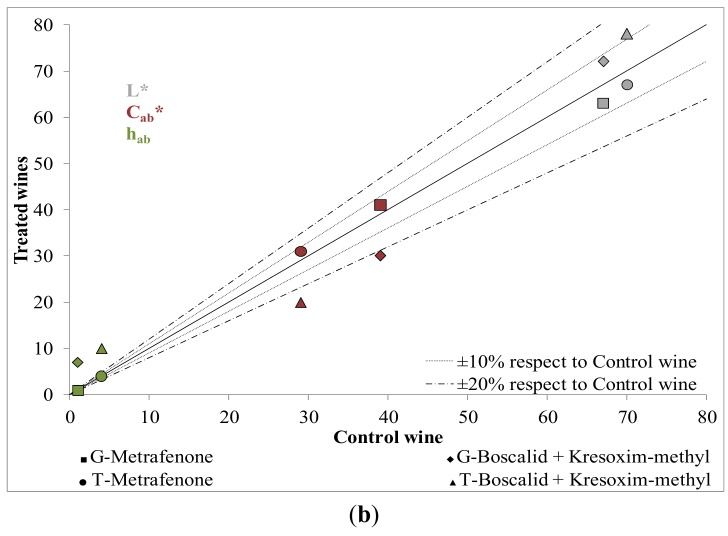
Colorimetric indexes (**a**) and CIELab data (**b**) of treated wines (Y axis) and control wines (X axis) for Graciano (G) and Tempranillo (T) wines.

In addition, the effect of tested fungicides on the color of T and G wines has been analyzed. The comparison between the colorimetric indexes in treated and control wines for T and G varieties can be observed in Figure 1a. Independent of the variety and fungicide treatment, the yellow, red and blue percentages in all treated wines varied less than ±10% with respect to the control. Nevertheless, the treatment with boscalid + kresoxim-methyl caused an increment in the tonality of wines of both varieties (87 in T and 74 in G wines) respect to their respective control wines (77 and 62). The comparison between CIELab coordinates (h_ab_, C_ab_* and L*) of treated wines respect to the control wines can be observed in the Figure 1b. Some marked differences (higher than 20%) were registered again in CIELab coordinates for those wines obtain from grapes treated with boscalid + kresoxim-methyl. These wines showed lower chroma and higher hue than their respective control wines and as a consequence, this fungicide treatment could promote wines with higher tonality and lower color vividness.

In order to know if the differences observed in the CIELab parameters represent chromatic changes that can be perceived by the human eye, the ΔE*_ab_ parameter (difference in color between treated and control samples in the CIELab space, calculated as the Euclidean distance between their location defined by L*, a* and b*) was calculated [9]. Changes in the CIELab parameters were more pronounced for boscalid + kresoxim-methyl treatments since ΔE*_ab_ parameter ranged from 10.7 to 11.5, while in wines treated with metrafenone the value was around 4.

### 2.2. Influence of New Generation Fungicides Residues on the Phenolic Profile of Wines

#### 2.2.1. Anthocyanins

Anthocyanins are the principal compounds responsible of the color of red wines. They are transferred from the skin of grapes to the wine during the early days of winemaking. Total anthocyanins of T and G wines and their distribution (% monomeric, % copigmented and % polymeric forms), determined by UV-Vis spectroscopy according to Boulton [11], can be seen in Table 1. The monomeric forms decreased in the first stages because they participate in the copigmentation process with other non- pigmented phenolic compounds, being flavonols the most effective copigments [12]. On the other hand, acetaldehyde, tannins and other phenolic compounds (catechins, proanthocyanidins), are involved in processes of condensation-polymerization with monomeric anthocyanins, leading to the formation of more stable polymeric pigments than the free monomeric forms [13,14,15]. For sample comparisons, a variability greater than 10% in the results determined in treated wines respect to the control was considered as a difference statistically significant (*p* < 0.05) according to the statistical treatment applied (*t*-Student test). 

**Table 1:** Anthocyanin composition and quantitative data (mg∙L^−1^ ± SD) of monomeric anthocyanins in Tempranillo and Graciano wines.

Wines	T-Control		T-Metrafenone		T-Boscalid-Kresoxim-methyl		G-Control		G-Metrafenone		G-Boscalid-Kresoxim-methyl	
**Anthocyanins by UV/Vis**												
Monomeric (%)	34.68 ± 0.949		38.91 ± 1.753 37.37 ± 1.594 23.71 ± 0.159 3.880 ± 0.014		28.83 * ± 1.689 53.66 * ± 0.740 17.51 * ± 0.949 4.100 * ± 0.057		29.58 ± 1.244 43.05 ± 1.054 27.37 ± 0.190 5.180 ± 0.028		38.20 * ± 2.645 39.48 ± 1.983 22.33 * ± 0.662 4.345 * ± 0.021		34.36 * ± 1.060 46.37 ± 0.994 19.27 * ± 0.066 4.270 * ± ≤0.001	
Copigmented (%)	41.87 ± 0.678											
Polymeric (%)	23.46 ± 0.271											
**TOTAL Anthocyanins (absorbance units)**	3.905 ± 0.021											
**Monomeric anthocyanins by HPLC**												
**Malvidin derivatives**												
malvidin-3-*O*-glucoside	161.59 ± 4.204 13.25 ± 0.886 5.79 ± 0.592 0.85 ± 0.021 0.54 ± 0.032 0.25 ± 0.005		152.12 ± 2.326 11.26 ± 0.018 5.05 ± 0.088 0.91 ± 0.039 0.56 ± ≤0.001 0.37 * ± 0.020		158.57 ± 4.068 13.46 ± 0.445 6.62 ± 0.058 1.55 * ± 0.023 0.47 ± 0.055 0.06 * ± 0.006		163.75 ± 1.684 8.87 ± 0.723 11.44 ± 0.382 1.40 ± 0.131 1.37 ± 0.049 0.46 ± 0.043		128.79 * ± 3.121 9.63 ± 0.783 10.93 ±1.116 1.62 ± 0.168 2.10 * ± 0.204 0.36 ± 0.004		121.94 * ± 4.149 7.82 ± 0.202 10.30 * ± 0.300 1.85 * ± 0.100 1.93 * ± 0.100 0.47± 0.040	
malvidin-3-*O*-(6-*O*-p-coumaroyl)glucoside												
malvidin-3-*O*-(6-*O*-acetyl)glucoside												
malvidin-3-*O*-(6-*O*-caffeoyl)glucoside												
vitisin A												
vitisin B												
**subTOTAL (mg∙L^−1^) **(%)	**182.12** (78.5)		**170.27** (79.9)		**180.73** (79.6)		**187.29** (80.8)		153.44 * (86.7)		144.31 * (85.6)	
**Petunidin derivatives**												
petunidin-3-*O*-glucoside	25.38 ± 1.947 3.00 ± 0.125 1.20 ± 0.117		22.82 ± 0.834 2.34 * ± 0.144 1.12 ± 0.059		25.32 ± 0.395 2.80 ± 0.059 1.00 ± 0.054		10.59 ± 0.095 0.39 ± 0.005 0.60 ± 0.045		6.35 * ± 0.074 0.03 * ± ≤0.001 0.60 ± 0.047		4.70 * ± 0.176 0.41 ± 0.020 0.27 * ± 0.016	
petunidin-3-*O*-(6-*O*-p-coumaroyl)glucoside												
petunidin-3-*O*-(6-*O*-acetyl)glucoside												
**subTOTAL (mg∙L^−1^) **(%)	**29.58** (12.7)		**26.28** (12.3)		**29.12** (12.7)		**11.58** (5.0)		6.98 * (3.9)		5.38 * (5.2)	
**Delphinidin derivatives**												
delphinidin-3-*O*-glucoside	10.06 ± 0.735 2.99 ± 0.111 0.66 ± 0.041		8.24 * ± 0.284 0.95 * ± 0.001 0.68 ± 0.017		11.06 ± 0.070 1.20 * ± 0.007 0.70 ± 0.015		5.08 ± 0.071 *n.d.* 0.37 ± 0.009		1.99 * ± 0.219 *n.d.* 0.32 ± 0.035		1.36 * ± 0.064 *n.d.* 1.36 * ± 0.156	
delphinidin-3-*O*-(6-*O*-p-coumaroyl)glucoside												
delphinidin-3-*O*-(6-*O*-acetyl)glucoside												
**subTOTAL (mg∙L^−1^) **(%)	**13.71** (5.9)		9.87 * (4.6)		**12.96** (5.6)		**5.45** (2.4)		2.31 * (1.3)		2.72 * (1.6)	
**Peonidin derivatives**												
peonidin-3-*O*-glucoside	4.53 ± 0.123 0.82 ± 0.023 0.06 ± 0.004*n.d.*		3.98 * ± 0.186 1.00 ± 0.010 0.37 * ± 0.004*n.d.*		4.69 ± 0.027 0.84 ± 0.082 0.10 * ± 0.009*n.d.*		18.76 ±0.385 4.39 ± 0.173 2.95 ± 0.053 0.16 ± 0.012		8.04 * ± 0.749 2.90 * ± 0.259 2.17 * ± 0.232 0.17 ± 0.008		10.39 * ± 0.290 2.49 * ± 0.060 2.30 * ± 0.222 0.25 * ± ≤0.001	
peonidin-3-*O*-(6-*O*-p-coumaroyl)glucoside												
peonidin-3-*O*-(6-*O*-acetyl)glucoside												
peonidin-3-*O*-(6-*O*-caffeoyl)glucoside												
**subTOTAL (mg∙L^−1^) **(%)	**5.41** (2.3)		**5.37** (2.5)		5.63 * (2.4)		**26.25** (11.3)		13.28 * (7.5)		15.43 * (9.1)	
**Cyanidin derivatives**												
cyanidin-3-*O*-glucoside	0.44 ± 0.003 0.56 ± 0.033 0.34 ± 0.017		0.48 ± 0.024 0.51 ± 0.002 0.41 * ± 0.011		0.46 ± 0.028 0.61 ± 0.031 0.38 * ± 0.004		0.43 ± 0.024 0.43 ± 0.006 0.22 ± 0.012		0.25 * ± 0.032 0.41 ± 0.021 0.34 * ± 0.001		0.25 * ± 0.017 0.10 * ± 0.009 0.49 * ± 0.007	
cyanidin-3-*O*-(6-*O*-p-coumaroyl)glucoside												
cyanidin-3-*O*-(6-*O*-acetyl)glucoside												
**subTOTAL (mg∙L^−1^) **(%)	**1.34** (0.6)		**1.40** (0.7)		**1.45** (0.6)		**1.09** (0.5)		**1.00** (0.6)		0.84 * (0.5)	
**TOTAL *monomeric anthocyanins* (mg∙L^−1^)**	**232.16**		**213.17**		**229.89**		**231.65**		177.01 *		168.68 *	

*: Statistical differences according to the *t*-student test (*p* < 0.05).

As can be seen in Table 1, the total anthocyanins content was lower in T wines than in G wines, although the percentages of monomeric, copigmented and polymeric forms were similar in both varieties (around 30%, 42% and 25%, respectively). Statistical tests confirmed a significant difference between control and wines treated with boscalid + kresoxim-methyl for both varieties; meanwhile the metrafenone treatment seems only to affect the anthocyanin percentages in G wines.

**Figure 2:**
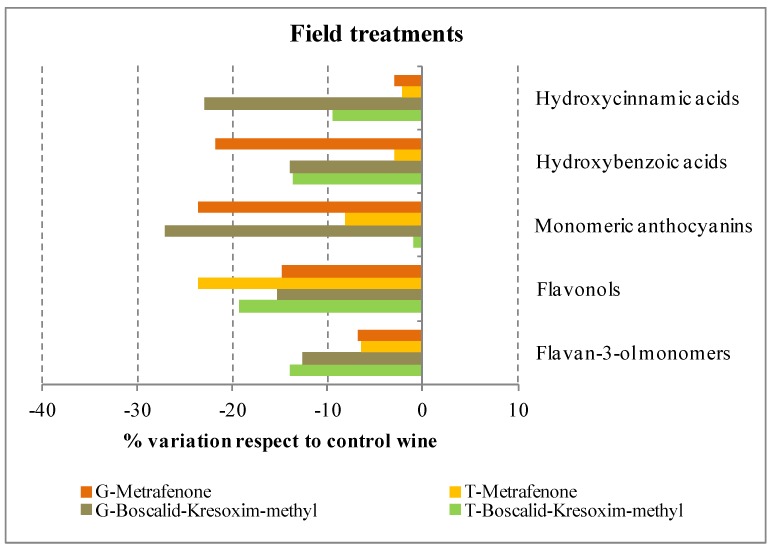
Effect of each antifungal treatment on the phenolic profile of Graciano (G) and Tempranillo (T) wines.

Monomeric anthocyanins content in T and G control wines, determined by chromatographic analysis, was similar (232.16 and 231.65 mg∙L^−1^, respectively), as can be seen in Table 1. However, fungicide treatments applied in the field affected the anthocyanin content differently, depending on the variety (as can be seen in Figure 2). Thereby, while for T wines no effects were observed, for G wines reductions of about 25% respect to the G-Control were observed for the two treatments. The most abundant anthocyanin compounds in both wines were malvidin derivatives (78.5% and 80.8% in T and G control wines, respectively), being malvidin-3-*O*-glucoside the most abundant anthocyanin in all wines (Table 1). Petunidin derivatives were the following most abundant derivatives for T wines (12.7%), being petunidin-3-*O*-glucoside the main compound in this group; in contrast, the second most abundant group for G wines was the peonidin derivatives (11.3%). Concentrations of the other minor derivative groups (ranging from 0.5% to 6%) were as follows: delphinidin > peonidin > cyanidin derivatives in T wines; meanwhile for G wines the minor groups were: petunidin > delphinidin > cyanidin derivatives.

#### 2.2.2. Flavan-3-ol Monomers and Proanthocyanidins

The total content of flavan-3-ol monomers was lower in T-Control than in G-Control (28.6 *versus* 49.6 mg∙L^−1^, respectively), as can be seen in Table 2. This is in agreement with other authors [16,17]. 

**Table 2:** Flavan-3-ol composition and quantitative data (mg∙L^−1^ ± SD) in Tempranillo and Graciano wines.

Wines	T-Control		T-Metrafenone		T-Boscalid-Kresoxim-methyl		G-Control		G-Metrafenone		G-Boscalid-Kresoxim-methyl	
**Flavan-3-ol monomers**												
catechin (C)	19.49 ± 0.013 7.93 ± 0.035 1.14 ± 0.023		19.26 ± 0.036 7.43 ± 0.239 *n.d.*		18.32 * ± 0.054 6.27 * ± 0.003 *n.d.*		24.39 ± 0.419 23.77 ± 0.270 1.48 ± 0.004		23.39 ± 0.276 21.70 ± 0.721 1.42 * ± 0.004		20.94 * ± 0.232 20.98 * ± 0.167 1.44 * ± 0.001	
epicatechin (EC)												
galocatechin (GC)												
**subTOTAL (mg∙L^−1^)**	**28.56**		**26.69**		24.59 *		**49.64**		**46.29**		43.36 *	
**Proanthocyanidins**												
aDP (%)	2.6 765.4 59 40 0.5		2.7 811.5 60 40 0.5		2.6 778.1 68 3 10.6		1.8 526.4 79 20 1.3		1.8 516.9 81 18 1.2		1.8 521.9 81 18 0.8	
aMW (%)												
procyanidins (%PC)												
prodelphinidins (% PD)												
galloylated (% G)												
**subTOTAL (mg∙L^−1^)**	**241.94 ± 0.11**		**228.05 ± 1.83**		200.80 * ± 1.11		**219.18 ± 1.46**		**207.51 ± 0.35**		191.40 * ± 2.23	

*: Statistical differences according to the *t*-student test (*p* < 0.05).

The main flavan-3-ol monomers in T and G wines were (+)-catechin (C), (−)-epicatechin (EC) and (−)-gallocatechin (GC) (Table 2). While the contents of C and EC in G-Control were similar, the content of EC in T-Control was about 40% of the C content. Significant differences were only observed for boscalid + kresoxim-methyl treatments, independent of the variety, with reductions of about 13% (see Figure 2).

In addition to flavan-3-ol monomers, there are also dimeric, trimeric, oligomeric and condensed procyanidins. These compounds are better extracted with longer maceration in the presence of alcohol [18]. We can find procyanidins —polymers of (epi)catechin that release cyanidin- and prodelphinidins —polymers of (epi)gallocatechin that release delphinidin [19]. This large group of compounds differs in the nature of their constitutive units, their number (degree of polymerization) and the position of linkages between them. All of the structures have not been analyzed, and only the procyanidin dimers and some of the trimers have been completely identified. 

After acid-catalyzed cleavage of the polymer in presence of phloroglucinol, the mean degree of polymerization (aDP, calculated as the ratio of total units to terminal units), the average molecular weight (aMW) and the total proanthocyanidins concentration (calculated as the sum of all units) were determined [19,20]. As it can be seen in Table 2, the concentration of total proanthocyanidins in T-Control was 241.94 mg∙L^−1^, slightly higher than that obtained in G-Control (219.18 mg∙L^−1^). In general, no important effects of metrafenone residues in the total proanthocyanidins content were observed (with reductions of around 5%) for T and G wines. Nevertheless the treatments with boscalid + kresoxim-methyl produced a reduction of over 15% for T and G wines. In addition, the aDP value obtained for T and G wines was 2.6 and 1.8, respectively, indicating that the proanthocyanidins present in these wines are mostly dimers. These results are in good agreement with those reported in similar wines by other authors [21].

#### 2.2.3. Flavonols

Flavonols were the minor flavonoid group with respect to the other flavonoid groups described above. As it can be seen in Table 3, flavonol content in T-Control was 8.20 mg∙L^−1^, being this concentration higher than that obtained in G-Control (6.46 mg∙L^−1^). Flavonol 3-*O*-glucoside derivatives are the main group contributing to the total flavonol content for T- and G-based wines. Five 3-*O*-glucoside derivatives (myricetin, quercetin, laricitrin, isohamnetin and syringetin) were detected in G wines, while kaempherol-3-*O*-glucoside was also detected in T wines. The concentrations of these derivatives ranged from 4.74 to 6.01 mg∙L^−1^ in T wines, myricetin-3-*O*-glucoside being the main flavonol; in G wines they ranged from 3.62 to 4.28 mg∙L^−1^, syringetin-3-*O*-glucoside being the most abundant. The next group is formed by the 3-*O*-glucuronide derivatives of myricetin, quercetin and kaempherol, with concentrations ranging between 1.07 and 1.37 mg∙L^−1^ in T wines, and between 1.12 and 1.43 mg∙L^−1^ in G wines. Furthermore, while in T-Control two galactoside derivatives were identified (quercetin and kaempherol), only quercetin-3-*O*-galactoside was detected in G-Control. Finally, four aglycone forms (myricetin, quercetin, kaempherol and laricitrin) were identified in both varieties in concentrations ranging from 0.35 to 0.60 and from 0.39 to 0.72 mg∙L^−1^ in T and G wines, respectively.

**Table 3:** Flavonol composition and quantitative data (mg∙L^−1^ ± SD) in Tempranillo and Graciano wines.

Wines	T-Control		T-Metrafenone		T-Boscalid-Kresoxim-methyl		G-Control		G-Metrafenone		G-Boscalid-Kresoxim-methyl	
**3-*O*-glucoside derivatives**												
myricetin-3-*O*-glucoside	3.46 ± 0.279 0.32 ± 0.021 1.01 ± 0.030 0.12 ± 0.001 0.15 ± 0.005 0.95 ± 0.025		2.48 * ± 0.072 0.36 * ± 0.021 0.78 * ± 0.035 0.09 * ± 0.002 0.13 * ± 0.004 0.90 ±0.078		2.50 * ± 0.215 0.26 * ± 0.012 0.86 * ± 0.028 0.14 * ± 0.009 0.14 ± 0.006 0.86 ± 0.057		0.61 ± 0.001 0.13 ± 0.002 0.74 ± 0.035 *n.d.* 0.25 ± 0.016 2.55 ± 0.161		0.37 * ± ≤0.001 *n.d.* 0.61 * ± 0.002 *n.d.* 0.24 ± 0.005 2.39 ± 0.072		0.37 * ± 0.001 0.13 ± 0.002 0.60 * ± 0.048 *n.d.* 0.44 * ± 0.022 2.36 ± 0.171	
quercetin-3-*O*-glucoside												
laricitrin-3-*O*-glucoside												
kaempherol-3-*O*-glucoside												
isohamnetin-3-*O*-glucoside												
syringetin-3-*O*-glucoside												
**subTOTAL (mg∙L^−1^) **(%)	**6.01** (73.3)		4.74 * (75.7)		4.76 * (71.9)		**4.28** (66.2)		3.62 * (65.8)		**3.90** (71.3)	
**3-*O*-glucuronide derivatives**												
myricetin-3-*O*-glucuronide	0.55 ± 0.006 0.63 ± 0.037 0.19 ± 0.017		0.43 * ± 0.040 0.50 * ± 0.029 0.14 * ± 0.001		0.41 * ± 0.024 0.63 ± 0.048 0.17 ± 0.001		0.23 ± 0.017 0.93 ± 0.038 0.23 ± ≤0.001		0.21 ± 0.007 0.95 ± 0.011 0.28 * ± 0.004		0.15 * ± 0.003 0.72 * ± 0.022 0.25 ± 0.001	
quercetin-3-*O*-glucuronide												
kaempherol-3-*O*-glucuronide												
**subTOTAL (mg∙L^−1^) **(%)	**1.37** (16.7)		1.07 * (17.1)		**1.21** (18.3)		**1.39** (21.5)		**1.43** (26.0)		1.12 * (20.5)	
**3-*O*-galactoside derivatives**												
quercetin-3-*O*-galactoside	0.14 ± 0.001 0.07 ± 0.001		0.10 * ± 0.001 *n.d.*		0.14 ± 0.003 0.08 ± 0.001		0.07 ± 0.002 *n.d.*		*n.d.* *n.d.*		0.06 * ± ≤0.001 *n.d.*	
kaempherol-3-*O*-galactoside												
**subTOTAL (mg∙L^−1^) **(%)	**0.21** (2.6)		0.10 * (1.6)		**0.22** (3.3)		**0.07** (1.1)				0.06 * (1.1)	
**Aglycons**												
myricetin	0.27 ± 0.025 0.21 ± 0.011 0.06 ± ≤0.001 0.07 ± ≤0.001		0.15 * ± 0.005 0.14 * ± ≤0.001 *n.d.* 0.06 * ± 0.002		0.23 ± 0.006 0.14 * ± 0.004 *n.d.* 0.06 * ±≤0.001		0.24 ± 0.019 0.37 ± 0.028 0.05 ± ≤0.001 0.06 ± 0.001		0.18 * ± ≤0.001 0.27 * ± ≤0.001 *n.d.* *n.d.*		0.11 * ± ≤0.001 0.17 * ± ≤0.001 0.05 ± ≤0.001 0.05 * ± ≤0.001	
quercetin												
kaempherol												
laricitrin												
**subTOTAL (mg∙L^−1^) **(%)	**0.60** (7.3)		0.35 * (5.6)		0.43 * (6.5)		**0.72** (11.1)		0.45 * (8.2)		0.39 * (7.2)	
**TOTAL flavonols (mg∙L^−1^)**	**8.20**		6.26 *		6.62 *		**6.46**		5.50 *		5.47 *	

*: Statistical differences according to the *t*-student test (*p* < 0.05).

**Table 4:** Phenolic acid composition and quantitative data (mg∙L^−1^ ± SD) in Tempranillo and Graciano wines.

Wines	T-Control		T-Metrafenone		T-Boscalid-Kresoxim-methyl		G-Control		G-Metrafenone		G-Boscalid-Kresoxim-methyl		
**Hydroxybenzoic acids**													
gallic acid	17.00 ± 0.788 6.01 ± 0.252 2.54 ± 0.098		17.25 ± 0.160 4.93 * ± 0.226 2.45 ± 0.124		13.88 * ± 0.754 5.69 ± 0.189 2.17 * ± 0.151		10.55 ± 0.046 3.42 ± 0.070 1.72 ± 0.106		5.41 * ± 0.110 2.42 * ± 0.120 1.58 ± 0.008		7.29 * ± 0.152 2.21 * ± 0.048 1.97 ± 0.003		
3,5-dihydroxibenzoic acid													
protocatechuic acid													
**Hydroxybenzoic acids**													
vanillic acid	2.58 ± 0.005 4.07 ± 0.007		3.19 ± 0.002 3.42 * ± 0.231		2.21 * ± 0.047 3.86 ± 0.297		6.25 ± 0.348 3.52 ± 0.355		6.23 ± 0.200 4.26 ± 0.134		5.93 ± 0.588 4.52 ± 0.393		
syringic acid													
**subTOTAL (mg∙L^−1^) **(%)	**32.20** (51.4)		**31.24** (51.2)		27.82 * (50.2)		**25.46** (65.2)		19.90 * (60.1)		21.91 * (67.9)		
**Hydroxycinnamic acids and their derivatives**													
caftaric acid	16.44 ± 1.022 0.46 ± 0.012 2.36 ± 0.141 10.08 ± 0.824 1.10 ± 0.112		16.48 ± 0.948 0.50 ± ≤0.001 2.14 ± 0.080 9.69 ± 0.631 0.98 ± 0.060		14.74 ± 0.478 0.32 * ± 0.001 2.13 ± 0.132 9.40 ± 0.645 0.98 ± 0.044		7.31 ± 0.130 0.12 ± 0.007 1.08 ± 0.003 3.17 ± 0.017 1.58 ± 0.127		7.37 ± 0.340 0.07 * ± 0.001 0.94 * ± 0.009 3.30 ± 0.192 1.20 * ± 0.017		5.77 * ± 0.487 0.01 * ± ≤0.001 0.78 * ± 0.034 2.37 * ± 0.228 1.30 * ± 0.073		
caffeic acid													
*c*-coutaric acid													
*t*-coutaric acid													
*p*-coumaric acid													
**subTOTAL (mg∙L^−1^) **(%)	**30.43** (48.6)		**29.78** (48.8)		**27.57** (49.8)		**13.27** (34.0)		**12.88** (38.9)		10.23 * (31.7)		
**Stylbene**													
resveratrol	*n.d.*		*n.d.*		*n.d.*		0.30 ± 0.002		0.32 ± 0.011		0.10 * ± 0.002		
**TOTAL phenolic acids (mg∙L^−1^)**	**62.63**		**61.02**		55.39 *		**39.03**		33.10 *		32.24 *		

*: Statistical differences according to the *t*-student test (*p* < 0.05).

Statistical differences in the flavonol profiles were observed for both treatments (metrafenone and boscalid + kresoxim-methyl) and varieties (Table 3) although greater reductions (around 20%–24%) were observed for T treated wines (Figure 2).

#### 2.2.4. Acids

The total phenolic acid content in T-Control was about 38% higher than that observed in G-Control. Stilbenes, hydroxybenzoic acids, hydroxycinnamic acids and their derivatives identified in T and G wines are listed in Table 4. Hydroxybenzoic acids content ranged between 27.82 and 32.20 mg∙L^−1^ in T wines, gallic and 3,5-dihydroxybenzoic acids being the main constituents among the five identified. Meanwhile, the content of these compounds in G wines comprised between 19.90 and 25.46 mg∙L^−1^, with gallic and vanillic acids being the most abundant compounds. Hydroxycinnamic acids (caffeic and coumaric acids) and their respective esters (caftaric and coutaric acid) were detected at concentrations between 27.57 and 30.43 mg∙L^−1^ in T wines, and between 10.23 and 13.27 mg∙L^−1^ in G wines, caftaric acid being the main compound in both varieties. In addition, the stilbene resveratrol was identified in G wines, but at low concentrations (between 0.10 and 0.32 mg∙L^−1^), representing less than 1% of total non-flavonoids. This compound was not detected in the wines obtained from T grapes. 

The different phytosanitary treatments had a variable effect in the accumulation of these compounds in the wines (Figure 2). In general terms, the treatments with metrafenone had no effect on the accumulation of hydroxycinnamic and hydroxybenzoic acids, except for G wines where a reduction of 22% respect to the control wine was observed. To the contrary, treatments with boscalid + kresoxim-methyl caused significant reductions for both acids and varieties, except for hydroxycinnamic acids in T treated wines.

## 3. Experimental

### 3.1. Fungicide Experiments

Fungicide experiments were performed out in 2012 at two experimental vineyards located in Aldeanueva de Ebro, La Rioja (N.E. Spain), belonging to D.O.Ca. Rioja. The vineyards produce *Vitis vinifera* cv. Tempranillo and cv. Graciano red grapes. The experimental vineyards were 3,000 m^2^ in area, approximately, and contained 30 rows with 40–50 vines each one; the gaps between rows and grapevines were 2.6 and 1.2 m, respectively. Each experimental vineyard was divided into three experimental plots: the first was untreated and used to produce the control wine, the other two were treated with the phytosanitary products Collis^®^ (BASF, 20% w/v boscalid + 10% w/v kresoxim-methyl) and Vivando^®^ (BASF, 50% w/v metrafenone), respectively, in accordance with good agricultural practices (GAP), using the doses recommended by the manufacturer and keeping the pre-harvest time in vines.

### 3.2. Winemaking Process and Wine Samples

The winemaking process was carried out in the experimental cellar located at the University of La Rioja. Identical vinifications were performed with the grapes from each experimental plot as follows: grapes were crushed, destemmed and placed in a metallic fermentation vessel (40 L) that was supplied with SO_2_ (at 50 mg∙L^−1^) concentration. The temperature during alcoholic fermentation–maceration, which took 14 days, was 17–21 °C. At the end of the process, the wine was strained off, grape residues were pressed and the wine–must mixtures were transferred to a metallic vessel where it was supplied with SO_2_ (at 30 mg∙L^−1^). Prior to bottling, a step of cold clarification was carried out.

### 3.3. Analytical Standards, Reagents and Materials

Malvidin-3-*O*-glucoside chloride, quercetin, catechin, epicatechin, resveratrol, and gallic, 3,5-dihydroxybenzoic, protocatechuic, vanillic, syringic, *p*-coumaric and caffeic acids were purchased from Sigma Aldrich (St. Louis, MO, USA). Individual stock solutions of each compound were prepared in methanol. Different working standards solutions were prepared by appropriate dilution in 12% ethanol in water and then stored in dark vials at −80 °C. Solvents (water, methanol, acetone and ethyl acetate) of HPLC grade and other inorganic reagents (formic, hydrochloric, acetic, trifluoroacetic and ascorbic acids, phloroglucinol, sodium acetate anhidro, and sodium bisulfite) were purchased from Sigma Aldrich. The sorbent materials used for SPE were: Oasis MCX cartridges (500 mg, 6 mL size) from Waters Corp (Milford, MA, USA); Strata-X-A 33u Polymeric Strong Anion sorbent (60 mg, 3 mL size) and Strata C18-E (2 g, 12 mL size) from Phenomenex (Torrance, CA, USA).

### 3.4. Characterization of the Color Fraction and Phenolic Content

The characterization of the color fraction was determined by spectrophotometric parameters, colorimetric indexes and CIELab parameters, using a Beckman Coulter DU 730 Life Science UV/Vis spectrophotometer. All of the measurements were carried out in duplicate, using quartz cells of 1 mm path length. A hydroalcoholic solution (12% ethanol) was used as blank in all measures.

*Colorimetric indexes**.*** Absorbances at 420, 520 and 620 nm were measured to assess the must color by chromatic parameters such as % red, % yellow and % blue, color intensity (CI) and tonality (T), according to Glories [22]. 

*CIELab coordinates**.*** The must color was also assessed by the CIELab space [23]. The parameters that define the CIELab space are: rectangular coordinates such as red/green color component (a*), yellow/blue color component (b*) and lightness (L*); and the cylindrical coordinates such as chroma (C_ab_*) and hue angle (h_ab_).

*Copigmented, monomeric, polymeric and total anthocyanins**.*** Each group of anthocyanins was determined according to Boulton [11]. Briefly, this method consisted of adjusting the pH of a wine to 3.6 and then filtering the wine through a 0.45 μm mesh filter. Then, the following tests were conducted:

- A^acet^: 20 μL of 10% (*v*/*v*) acetaldehyde was added to 2 mL of prepared wine and the sample was allowed to sit for 45 min at room temperature before measuring A_520 nm_;
- A^2^^0^: to another 100 µL of prepared wine, 1,900 µL hydroalcoholic solution was added and absorbance A_520 nm_ was also measured;
- A^S^^O2^: 160 μL of 5% (w/v) SO_2_ was added to 2 mL of prepared wine and absorbance A_520 nm_ was measured.

From these readings, the different forms of anthocyanins were expressed in absorbance units as:

copigmented anthocyanins = A^acet^ − A^2^^0^
monomeric anthocyanins = A^2^^0^ − A^S^^O2^
polymeric anthocyanins = A^S^^O2^
total anthocyanins = A^acet^

The percent distribution of the various forms was calculated as:

% copigmented = [(A^acet^ − A^2^^0^)/A^acet^] × 100
% monomeric = [(A^2^^0^ − A^S^^O2^)/A^acet^] × 100
% polymeric = [A^S^^O2^/A^acet^] × 100

### 3.5. Determination of Phenolic Compounds

#### 3.5.1. Extraction Procedures

***Flavan-3-ol monomsers and proanthocyanidins.*** Proanthocyanidins were extracted and characterized according to the procedure described by Kennedy and Jones [24], with minor modifications [20]. Briefly, bleaching of anthocyanins pigments is necessary prior to retained proanthocyanidins by anion exchange sorbent. After eluting with 75% acetone in water, proanthocyanidins were acid-catalyzed in presence of phloroglucinol. This process followed by HPLC analysis is a useful alternative for quantification and characterization of longer proanthocyanidins.

*(a) Flavan-3-ol monomers and proanthocyanidins* *extraction.* Wine (2 mL) was adjusted to pH 1.0 with a drop of concentrated hydrochloric acid, transferred to a 5 mL test tube containing sodium bisulfite (800 mg) and stirred for 20 min. Under these conditions, most of monomeric anthocyanins are combined with bisulfite to form colorless sulfonic acid adducts [25], which can be readily retained by anion exchange sorbents. This bleached wine was diluted 1:2 with ultrapure water and an aliquot (2 mL) was loaded into a Strata-X-A mixed-mode anion exchange/reversed phase SPE cartridge, previously activated with 75% acetone in water (2 mL) followed by water (4 mL). Afterwards, the cartridge was washed with water (4 mL) and flavan-3-ols and proanthocyanidins were eluted with 75% acetone in water (8 mL), whereas anthocyanins and organic acids were still retained through anion exchange interactions. This eluate was brought to dryness on a rotary evaporator at 35 °C and then reconstituted in methanol (200 µL). In order to quantify monomeric flavan-3-ols, a portion of this methanolic extract (50 µL) were diluted to 500 µL with 2.5% acetic acid in water, filtered (0.20 µm) and analyzed by HPLC/DAD–ESI/MS.

*(b) Acid-catalyzed degradation of proanthocyanidins* *in presence of phloroglucinol.* Proanthocyanidins were characterized following the acid-catalyzed cleavage of the polymer in the presence of phloroglucinol excess according to the procedure described by Kennedy and Jones [24] with minor modifications. In brief, a solution containing 0.2 M HCl, 50 mg∙mL^−1^ phloroglucinol and 10 mg∙mL^−1^L-ascorbic acid was prepared in methanol as the phloroglucinolysis reagent. Methanolic wine extract (100 µL) was allowed to react with phloroglucinol solution (200 µL) in a water bath for 40 min at 50 °C. Afterwards, the reaction was cooled down and quenched by the addition of 15 mM sodium acetate aqueous solution (2.7 mL). The reaction mixture was then purified by SPE using a Strata-X-A cartridge SPE previously conditioned with 75% acetone in water (2 mL) followed by water (4 mL). The cartridge was washed with water (4 mL) and the phloroglucinolysis products were eluted with 75% acetone in water (8 mL). This eluate was evaporated to dryness on a rotary evaporator at 35 °C, reconstituted in 2.5% acetic acid in water (1 mL), filtered (0.20 µm) and analyzed by HPLC/DAD–ESI/MS.

***Anthocyanins.*** Wine samples were previously evaporated under a stream of nitrogen to remove ethanol and reconstituted with water. A sample of the reconstituted wine (2 mL) was loaded onto a Strata C18 cartridge, previously activated with methanol (10 mL) followed by water (10 mL). The sorbent was dried by blowing N_2_ for 30 min. After washing with ethyl acetate (20 mL), the anthocyanin fraction was eluted with 0.1% TFA in methanol (30 mL). The eluate was evaporated to dryness (35 °C, 10 psi) and redisolved in 12% ethanol in water (1 mL). The ethanolic extract was passed through a filter of 0.45 µm pore size prior to HPLC/DAD–ESI/MS analysis.

***Phenolic acids, resveratrol and flavonols.*** Wine samples were previously evaporated under a stream of nitrogen to remove ethanol and reconstituted with water. The reconstituted wine (3 mL, adjusted to pH 7) was loaded into a MCX cartridge previously activated with methanol (5 mL) followed by water (5 mL). The sorbent was washed with 0.1 M hydrochloric acid (5 mL) followed by water (5 mL). The acid and flavonol fractions were eluted with methanol (15 mL). The eluate was evaporated to dryness (35 °C, 10 psi) and redissolved in 12% ethanol in water (1 mL). The ethanolic extract was passed through a filter of 0.45 µm pore size prior to HPLC/DAD–ESI/MS analysis.

#### 3.5.2. HPLC/DAD–ESI/MS Analysis

Identification of these groups of polyphenols was performed according to the HPLC/DAD-ESI/MS procedures described by Figueiredo-González *et al.* [26] and Quijada-Morín *et al.* [20]. HPLC measurements were made by using a Thermo Separation-Products (TSP, Waltham, MA, USA) system comprised of a P2000 binary pump equipped with a TSP AS1000 autosampler, and a TSP SCM1000 vacuum membrane degasser. An analytical column, Phenomenex C18 Luna (150 × 3 mm i.d., 5 µm), with a guard column, Pelliguard LC-18 (50 × 4.6 mm i.d., 40 µm; Supelco, Bellefonte, PA, USA) was used for separation of anthocyanins, phenolic acids, resveratrol and flavonols and other analytical column, Phenomenex C18 Luna (150 × 3 mm i.d., 3 µm) was used for separation of flavan-*3*-ol monomers and proanthocyanidins. UV–Vis spectra were scanned from 200 to 600 nm on a diode array UV6000LP DAD detector. For confirmation purposes, the HPLC–DAD system was coupled to a TSQ Quantum Discovery triple-stage quadrupole mass spectrometer from Thermo Fisher Scientific (Waltham, MA, USA). The mass spectrometer was operated in the negative electrospray ionization (ESI) mode under the following specific conditions: spray voltage 4,000, capillary temperature of 250 °C, sheath gas and auxiliary gas pressure of 30 and 10 units, collision energy 25 and tube lens offset 110. The detection was accomplished in the full-scan mode, from *m/z* 100 to 1,700, and in the MS/MS mode.

*Flavan-3-ol monomers and proanthocyanidins.* Acetic acid extract (20 µL) was injected into the column and eluted at 30 °C. Mobile phase A and B were 0.1% formic acid aqueous solution and 95% acetonitrile (in 5% mobile phase A) respectively, and the flow rate was 0.4 mL∙min^−1^. The linear gradient used was as follows: 0–2 min, 98% A and 2% B; 20–22 min, 90% A and 10% B; 50 min, 85% A and 15% B; 60 min, 80% A and 20% B; 70 min, 60% A and 40% B; 72–80 min, 10% A and 90% B; 82–92 min, 98% A and 2% B. DAD chromatograms were registered at 280 nm.

Due to the lack of the corresponding standards, extension subunits, *i.e.*, flavan-3-ol phloroglucinol adducts, were quantified using their molar response factors relative to catechin as reported by Kennedy and Jones [24]. In any case, the presence of the phloroglucinol adducts was confirmed by mass spectrometry. The mass spectrum of the gallocatechin phloroglucinol adduct obtained in the ESI negative mode exhibited a [M−H]^−^ ion at *m/z* 429 and a [2M−H]− ion at *m/z* 859. MS/MS fragmentation of *m/z* 429 produced a daughter ion at *m/z* 303 [M−H−C_6_H_6_O_3_]^−^, which was indicative for a loss of phloroglucinol (126 Da) and the retro-Diels-Alder (RDA) product at *m/z* 261 [M−H−C_8_H_8_O_4_]^−^. The MS analysis of catechin and epicatechin adducts showed a [M−H]^−^ ion at *m/z* 413 and a [2M−H]− ion at *m/z* 827. MS/MS fragmentation product ions of *m/z* 413 were detected at *m/z* 287 [M−H−C_6_H_6_O_3_]^−^ (loss of phloroglucinol) and at *m/z* 261 [M−H−C_8_H_8_O_3_]^−^ (RDA fission).

*Anthocyanins.* Ethanolic extract (20 µL) was injected into the column and eluted at 35 °C. Mobile phase A and B were 5% formic acid aqueous solution and methanol, respectively, and the flow rate was 1 mL∙min^−1^. The following linear gradient was used: 0–5 min, 90% A and 10% B; 15 min, 80% A and 20% B; 30 min, 70% A and 30% B; 40–85 min, 68% A and 32% B; 87 min, 60% A and 40% B; 96 min, 50% A and 50% B; 98–108 min, 5% A and 95% B; 110–120 min, 90% A and 10% B. DAD chromatograms were registered at 520 nm.

*Phenolic acids and resveratrol.* Ethanolic extract (20 µL) was injected into the column and eluted at 35 °C. Mobile phase A and B were 0.2% formic acid aqueous solution and methanol, respectively, and the flow rate was 0.8 mL∙min^−1^. The following linear gradient was used: 0 min, 97% A and 3% B; 40 min, 70% A and 30% B; 50–53 min, 50% A and 50% B; 55–65 min, 5% A and 95% B; 67–77 min, 97% A and 3% B. DAD detection wavelengths of 280, 320 and 309 nm were selected for phenolic acids, hydroxycinnamic acids and resveratrol, respectively.

*Flavonols.* Ethanolic extract (20 µL) was injected into the column and eluted at 35 °C. Mobile phase A and B were 2.5% formic acid aqueous solution and methanol, respectively, and the flow rate was 1 mL∙min^−1^. The following linear gradient was used: 0 min, 80% A and 20% B; 10 min, 75% A and 25% B; 30 min, 65% A and 35% B; 40–42 min, 60% A and 40% B; 45 min, 50% A and 50% B, 48 min, 40% A and 60% B; 50–60 min, 5% A and 95% B; 62–72 min, 80% A and 20% B. DAD chromatograms were registered at 370 nm.

### 3.6. Statistical Analysis

For sample comparison, the data are presented as means ± standard deviation (SD) of analyses performed in triplicate. Significant differences among treated wines and control wines for each variety and compound were assessed by the *t*-student test. Data analyses were performed using the STATGRAPHICS Centurion XV 15.2.05 Software (Statpoint Technologies, Inc., Warrenton, VA, USA). 

## 4. Conclusions

Results showed that the wines obtained from grapes treated under good agricultural practices with boscalid + kresoxim-methyl had lower chroma and higher hue than control wines, resulting in less colorful wines. The ΔE*_ab_ parameter confirmed that these CIELab changes could be perceived by wine consumers. Although significant differences were observed for all determined phenolic compounds in wines, for both treatments and varieties, the results showed again that G and T wines obtained from grapes treated with boscalid + kresoxim-methyl were the most affected.
